# Hypotheses, Limits, Models and Life

**DOI:** 10.3390/life5010001

**Published:** 2014-12-29

**Authors:** William Bains

**Affiliations:** Rufus Scientific Ltd., 37 The Moor, Melbourn, Royston, Herts SG8 6ED, UK; E-Mail: bains@mit.edu

## Abstract

*Life* is launching a new section, called *Hypotheses in the Life Sciences*. The new Section will complement the other sections of *Life*, providing a feedstock of ideas whose tests can be published in the wider *Life* family, and elsewhere. We will consider hypotheses that are supported by real world, rigorous evidence, by clear arguments, and which provide a potential solution to a genuine gap in our understanding of any aspect of the life sciences.

This editorial marks the launch issue for a new section in MDPI’s journal *Life*, called *Hypotheses in the Life Sciences*. This is a continuation of a project to publish well-argued, well-supported hypotheses that was started with a smaller, independent journal [1,2]. We plan this Section to be a complement for the other sections of *Life*, providing a feedstock of ideas whose tests can be published in the wider *Life* family, and elsewhere. Prof. Rampelotto’s inaugural editorial as Editor in Chief of *Life* emphasized the importance of hypothesis as a driver for advances in science [3], and the new section is a concrete implementation of that importance.

A hypothesis is not “theory”, the dense, technical working out of a rigorous model. A hypothesis is the suggestion of a new explanation of existing facts, which subsequent theoretical and empirical work will develop to something robust enough to be called a theory. Of course, the Internet is full of hypotheses, with vehemently argued blogs about how the Moon landings were faked, dinosaur skeletons are an atheist conspiracy, and how almost every substance in existence causes (or cures) cancer [4]. These are not hypotheses in which we are interested. We are interested in hypotheses that are supported by real world, rigorous evidence, by clear arguments, and which provide a potential solution to a genuine gap in our understanding of the world. Such hypotheses often present a new model for understanding a system, and new models lead to new tests. There are very few places where such ideas can be published [3], but they have value [5,6] as they can stimulate experimentalists to look at their chosen system in a new way, to ask new questions, to look for new data. 

I have discussed before how I think a good hypothesis should be described [7,8]. To summarise, a hypothesis should explain something that needs explaining, should take account of what is already known, and should lay out a clear, logical and compelling argument. Specific facts may be discarded, but only for a reason. We know that much of the biomedical literature is flawed [8], but the argument that goes:

- Fact X does not fit my hypothesis
- Some facts are wrong
- Therefore I will assume that Fact X is wrong

simply will not do.

Any paper submitted to the new section will be peer reviewed, but our object is not just to find an excuse to block the publication of new or challenging ideas. Peer review can, and often is, a block to heterodoxy and innovation [9]. Both Prof. Rampelotto [3] and I intend to use peer review as a tool to ask “What will the readers think of this?”. If your paper cannot convince even one informed reader that your hypothesis is right, if two or three informed readers point out glaring gaps or errors in your argument, or if they throw up their hands in despair and say “I cannot understand any of this”, then either there is something wrong with the hypothesis or there is something wrong with your description of the hypothesis. In either case, your paper needs revision.

The Hypotheses in the Life Sciences section will publish papers on any aspect of the life sciences, providing the hypothesis either suggests a solution to a particularly important problem or suggests an idea with fairly wide relevance to the life sciences. A hypothesis that is of interest to only five people out of the 7 billion people on the planet is likely to be rejected.

We will launch the new Section with a special issue dedicated to the physicochemical limits of life, co-edited by noted microbiologist Prof. John Baross. We cannot experimentally prove the limits of life, as a limit is that beyond which no life can survive, and proving an absence is impossible. (Are you *sure* there are no bacteria that can grow at 123 °C rather than 122 °C [10]? None? Anywhere?) But we can hypothesise what the nature of the limits are, based on sound understanding of physics, chemistry and biology, and so this is fertile ground for hypotheses. It is also important. The limits of life have profound implications for the nature and origin of life, and for the possibility of life on other worlds. As we discover Super-Earth’s—planets more massive than Earth that are likely to have a rocky surface—we start to ask whether life can survive at gigapascal pressures or 200 °C temperatures. But the limits also have practical implications for understanding our own world. How deep can rock-dwelling microorganisms penetrate into our own planet to modify its geochemistry? Is every soil on the planet a potential sink for atmospheric carbon, or are some inherently uninhabitable? These are questions with real-world implications.

We hope you find the *Hypothesis in the Life Science* section of *Life* useful, stimulating and worthwhile to read, and we look forward to publishing your new hypotheses here.
